# Editorial: Surgical Advances in Pancreaticobiliary Diseases

**DOI:** 10.3390/jcm12041268

**Published:** 2023-02-06

**Authors:** Kota Sahara

**Affiliations:** 1 Department of Gastroenterological Surgery, Yokohama City University School of Medicine, Yokohama 236-0004, Japan; s.kota90@gmail.com; 2 Division of Surgical Oncology, James Comprehensive Cancer Center, Wexner Medical Center, Department of Surgery, The Ohio State University, Columbus, 43210 OH, USA

Pancreaticobiliary diseases include malignant tumors arising in organs with a complex anatomy, such as the pancreas and bile ducts, often presenting as locally advanced or metastatic lesions, and they frequently have a poor prognosis. Particularly, pancreatic malignancy is represented by pancreatic ductal adenocarcinoma (PDAC) and pancreatic neuroendocrine tumor (pNET), while biliary tract cancers (BTC) consist of intrahepatic (ICC) and extrahepatic cholangiocarcinoma (ECC) such as hilar cholangiocarcinoma and gallbladder cancer. Furthermore, pancreaticobiliary malignancy is on the rise, and pancreatic cancer is estimated to surpass breast, prostate, and colorectal cancers as the second leading cause of death by 2030 [1]. In the same manner, an increase in the incidence of cholangiocarcinoma has been reported worldwide, with the age-adjusted incidence of intrahepatic cholangiocarcinoma in the United States reported to have increased from 0.44 to 1.18 per 100,000 persons in the last 30 years [2,3].

In addition, pancreaticobiliary malignancies are known for their dismal prognosis. Patients with advanced gallbladder cancer and ECC who undergo resection have a poor 5-year prognosis ranging from 10 to 25% and 2 to 30%, respectively [4,5]. A separate study from our group found that roughly 1 in 5 patients with ICC had recurrence within six months following curative-intent resection [6]. As a result, surgeons and clinicians are still facing difficulties in treating pancreaticobiliary malignancies, and new techniques and therapeutic strategies are needed [7].

The current Special Issue, “Surgical Advances in Pancreaticobiliary Diseases” in the *Journal of Clinical Medicine* is dedicated to collecting high-quality scientific contributions that mainly focus on modern surgical techniques and treatment strategies.

In recent decades, robotic and laparoscopic pancreaticobiliary surgery has been increasingly utilized due to its multiple advantages. Indeed, several studies noted that minimally invasive surgeries for pancreaticobiliary diseases are associated with a shorter hospital stay, reduced blood loss, and equivalent complication rates compared with open surgery [8,9]. In the past, reports on laparoscopic surgery mainly focused on benign diseases and borderline malignancies, but in recent years, there have been studies on malignant tumors. Bauman et al. noted that long-term oncologic outcomes including distant or local recurrence and median survival were similar between laparoscopic (LDP) and open distal pancreatectomy for pancreatic cancer [10]. However, LDP for pancreatic cancer is mostly performed in high-volume centers, and its usefulness in cases with invasion of other organs or those requiring combined vascular resection remains unknown. Moreover, the safety of minimally invasive surgery for BCT is also unclear. Several meta-analyses have reported that laparoscopic hepatectomy of ICC was associated with better short-term (e.g., hospital stay and morbidity) and long-term outcomes (e.g., tumor recurrence), whereas those studies could not avoid considerable bias of patient and tumor factors related to the retrospective study [11,12].

One of the advances in the surgical field is the expansion of the limits of curative surgical resection for pancreaticobiliary cancers. Particularly, patients with locally advanced or distant metastatic pancreaticobiliary cancers have increased chances of receiving resection by a combination of neoadjuvant therapy and conversion surgery, resulting in relatively high R0 resection rates and favorable prognoses versus unresectable diseases [5,13,14,15]. A previous study by Suker and colleagues assessed the impact of FOLFIRINOX for locally advanced pancreatic cancer in a systematic review [16]. In this study, roughly 1 in 3 patients (28%) underwent resection after FOLFIRINOX, and the R0 resection rate ranged from 50% to 100% [16]. Surgical strategies such as distal pancreatectomy with celiac axis resection (DP-CAR) could also be performed to achieve a curative-intent resection for patients with locally advanced pancreatic cancer. While DP-CAR is useful as a curative procedure for locally advanced pancreatic cancer, the rates of acute renal failure and mortality were reported to be approximately ten times higher than those of conventional DP [17]. Hence, a multi-institutional study developed a novel risk prediction model to estimate a 90-day mortality risk after DP-CAR, as well as to help clinician appropriately select surgical candidates [18]. Thus, future studies are needed to compare the possibility of curative treatment of advanced pancreaticobiliary cancer with the increasing risk of postoperative morbidity and mortality. In settings of BTC, increasing literature studies have discussed the efficacy and safety of hepatopancreatoduodenectomy (HPD) in patients with locally extended BTC; however, the postoperative mortality rate was reported to be as high as 10–20% [19,20,21]. Given the very high risk of operative morbidity and mortality, there has been increasing interest in defining who should benefit from HPD. As such, these surgical advances combined with perioperative chemoradiotherapy have steadily contributed to a better prognosis of patients with pancreaticobiliary diseases, but many issues still remain to be resolved. 

In conclusion, the knowledge and application of modern technology and the continuous search for pancreaticobiliary diseases are important to treat patients with pancreaticobiliary tumors more safely and efficiently.

As a Guest Editor, I would like to sincerely thank the reviewers for their insightful remarks and the *JCM* team’s support. Finally, we heartily thank all of the contributing authors for their valuable input.

## Data Availability

No new data were created or analyzed in this study. Data sharing is not applicable to this article.
